# Reliability of intraoperative visual evoked potentials (iVEPs) in monitoring visual function during endoscopic transsphenoidal surgery

**DOI:** 10.1007/s00701-023-05778-1

**Published:** 2023-09-21

**Authors:** Pier Paolo Mattogno, Quintino Giorgio D’Alessandris, Mario Rigante, Giuseppe Granata, Michele Di Domenico, Valerio Perotti, Nicola Montano, Martina Giordano, Sabrina Chiloiro, Francesco Doglietto, Alessandro Olivi, Liverana Lauretti

**Affiliations:** 1 Institute of Neurosurgery, Fondazione Policlinico Gemelli IRCCS, Università Cattolica del Sacro Cuore - Roma, Largo A. Gemelli 8, 00168 Rome, Italy; 2 Department of Otorhinolaryngology, Fondazione Policlinico Gemelli IRCCS, Università Cattolica del Sacro Cuore - Roma, Largo A. Gemelli 8, 00168 Rome, Italy; 3 Department of Neurology, Fondazione Policlinico Gemelli IRCCS, Università Cattolica del Sacro Cuore - Roma, Largo A. Gemelli 8, 00168 Rome, Italy; 4 Department of Anesthesiology, Fondazione Policlinico Gemelli IRCCS, Università Cattolica del Sacro Cuore - Roma, Largo A. Gemelli 8, 00168 Rome, Italy; 5 Pituitary Unit, Department of Endocrinology and Metabolism, Fondazione Policlinico Gemelli IRCCS - Università Cattolica del Sacro Cuore - Roma, Largo A. Gemelli 8, 00168 Rome, Italy

**Keywords:** Endoscopic endonasal surgery, Visual evoked potentials, Neuromonitoring, Pituitary adenoma, Transsphenoidal

## Abstract

**Objective:**

To refine a reliable and reproducible intraoperative visual evoked potentials (iVEPs) monitoring protocol during endoscopic transsphenoidal surgery. To assess the reliability of baseline iVEPs in predicting preoperative visual status and perioperative iVEP variation in predicting postoperative visual outcome.

**Methods:**

Sixty-four patients harboring tumors of the pituitary region were included. All patients underwent endoscopic endonasal approach (EEA) with iVEPs monitoring, using a totally intravenous anesthetic protocol. Ophthalmological evaluation included visual acuity and visual field studies.

**Results:**

Preoperatively, visual acuity was reduced in 86% and visual field in 76.5% of cases. Baseline iVEPs amplitude was significantly correlated with preoperative visual acuity and visual field (*p* = 0.001 and *p* = 0.0004, respectively), confirming the reliability of the neurophysiological/anesthetic protocol implemented.

Importantly, perioperatively the variation in iVEPs amplitude was significantly correlated with the changes in visual acuity (*p* < 0.0001) and visual field (*p* = 0.0013). ROC analysis confirmed that iVEPs are an accurate predictor of perioperiative visual acuity improvement, with a 100% positive predictive value in patients with preoperative vision loss.

**Conclusions:**

iVEPs during EEA is highly reliable in describing preoperative visual function and can accurately predict postoperative vision improvement.

**Significance:**

iVEPs represent a promising resource for carrying out a more effective and safe endoscopic transsphenoidal surgery.

**Supplementary Information:**

The online version contains supplementary material available at 10.1007/s00701-023-05778-1.

## Introduction

Visual deterioration is the most common neurological sign of an enlarging mass in the pituitary region: during tumor removal, monitoring visual function to detect promptly worsening or amelioration would help the surgeon in deciding the most appropriate conduction.

Visual disturbances are caused by tumor compression on the different portions of the optic pathways but may also be the consequence of surgical tumor removal. In fact, a direct damage may follow the dissection of a stiff tumor from the optic structures while an indirect injury may occur by coagulating its vascular supply. The endonasal endoscopic approach (EEA) and its extended variant are the gold standard for the surgical treatment of pathologies of the hypothalamic-pituitary region: enhanced visualization and magnification of the anatomical structures facilitate a more complete tumor removal. Then, a more accurate neurophysiological monitoring is warranted for a safe surgery [1, 6, 32].

To achieve this goal, the monitoring of intraoperative visual evoked potentials (iVEPs) is a possible resource: in the last few years, there have been reports on its clinical application, but more data are necessary to assess reliability and predictive value. In fact, differently from SSEP and MEP, VEPs present a high intra-individual variability and relative instability/susceptibility of the acquisitions, limiting their wide clinical use [4, 8, 14, 15, 17, 19, 22, 28].

On this basis, a novel VEP acquisition protocol was established and reproduced for all patients included in the study, performing TIVA anesthesia, using a short duration, high intensity white light LED flash stimulus to optimize visual response evocation, and monitoring with electroretinogram and electroencephalogram, respectively, the visual stimulus and the correct neuroanaesthesiological level. As far as is known, not similar VEP protocols are reported in the literature.

For these reasons, the aims of the present study are (1) to develop a reliable and reproducible protocol for iVEPs in EEA; (2) to verify the reliability of baseline iVEPs in describing the preoperative visual status; and (3) to verify the reliability of the iVEPs in predicting the postoperative visual outcome.

## Methods

A prospective study was conducted to evaluate the feasibility and reliability of iVEPs monitoring on patients undergoing EEA for the treatment of sellar-suprasellar lesions, in a single tertiary institution. This study was approved by the Institutional Ethics Committee (Prot. 17814/19, ID: 2557). Enrolled patients signed an informed consent form; consent for publication of protected health information was obtained.

### Enrollment and inclusion criteria

We included in the study all consecutive patients operated upon for sellar-suprasellar lesions with clinical and radiological evidence of optic pathways impairment in the period October 2019–July 2021. Patients < 18 years of age and patients with primary or secondary blindness were excluded.

### Baseline preoperative evaluation

All patients underwent to preoperative ophthalmological evaluation (visual acuity with Snellen chart and visual field test with computerized perimetry).

### Intraoperative visual evoked potentials (iVEPs)

**Fig. 1:**
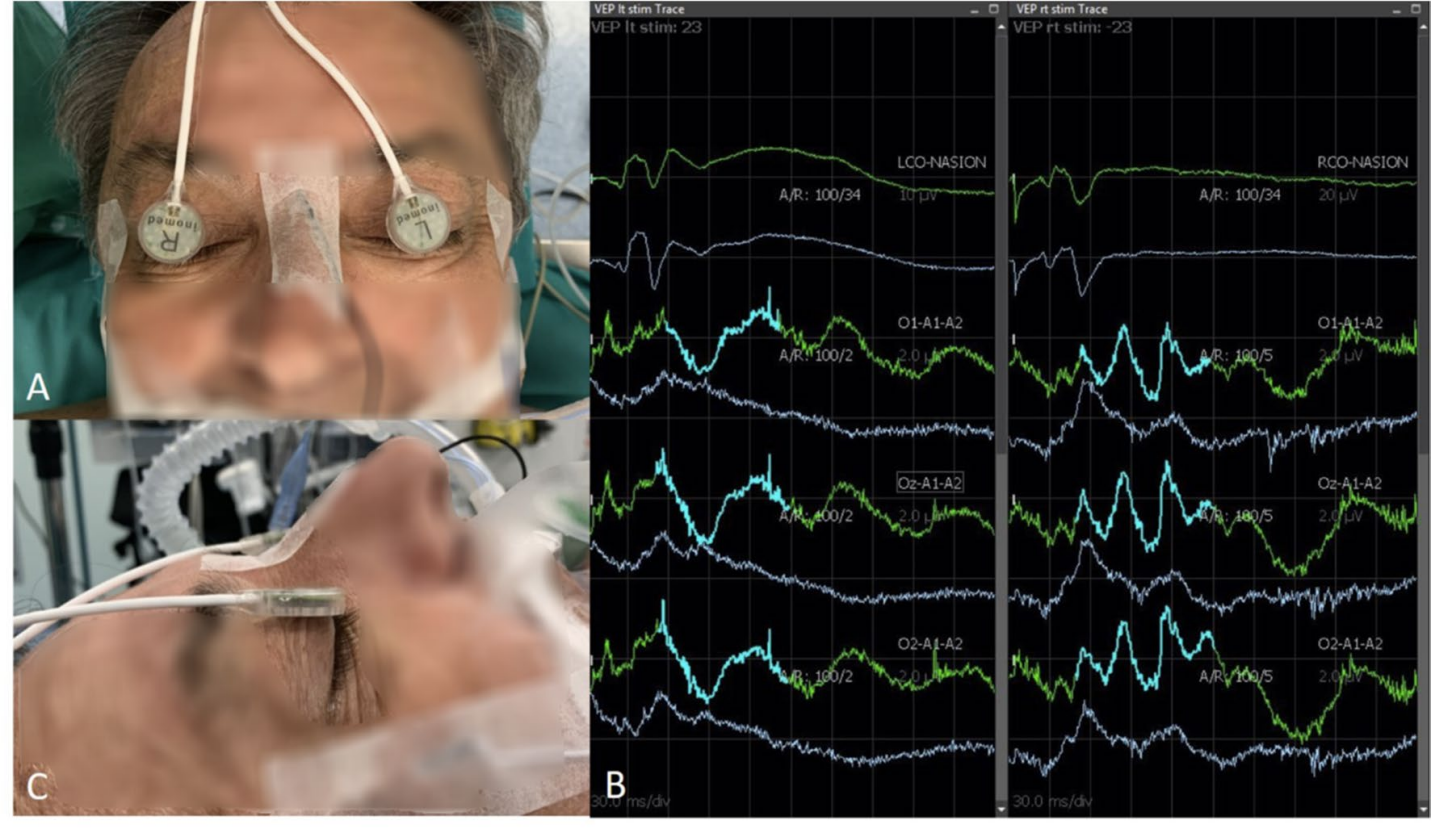
Setting of the iVEPs monitoring including ERG and EEG (**A**, **C**); iVEP acquisition. Two columns represent left and right eyes; from above ERG, iVEPs in O1, Oz, and O2: in grey the basal traces, in green with a wave highlighted in blue the variations during surgery (**B**)

iVEPs monitoring was carried out with white light LED flash stimulation delivered by 3 LEDs with a power of 5500 lx each (total power 16,500 lx). The hardware used was the Medtronic NIM-ECLIPSE® E4 and the stimulation was performed with Inomed LED flash goggles in a mono and binocular manner, fixed on the closed eyelids with adhesive plastic and plaster (Fig. 1). iVEPs were acquired at regular intervals as described in this paragraph. The duration of the stimulus was 1 ms and the stimulation frequency 1.1 Hz. The recording was performed by using corkscrew electrodes, positioned in O1, O2, and Oz position according to the 10–20 international system with a biauricular reference (A1 + A2). The acquisition time was 300 ms with an average of at least 100 traces to reduce the artefactual component. iVEPs component studied is the N2-P2 [20]. N2-P2 is the second negative and positive peak, respectively. The latency of this wave is near 100 ms and is considered as the P100 of the pattern visual evoked potentials. Bilateral ERG was performed to be sure of retinal activation and to avoid false positive responses since a consensual reduction or loss of ERG and iVEPs suggests a technical problem [16]. Stimulation parameters of ERG were the same of iVEPs while recording electrodes were positioned on the ipsilateral, lateral cantus oculi (referred to nasion).

iVEPs recording was performed at different steps as follows:

1. Baseline iVEPs: acquired under anesthesia, after disinfection and surgical dressing, before entering the endoscope into the nostrils.
2. Perioperative iVEPs: during the surgical procedure, from the opening of the sellar dura iVEPs were acquired regularly at 5′ intervals, marking down the most significant steps as “dura opening,” “tumor debulking,” “tumor capsule dissection,” “sellar reconstruction,” and any other surgical maneuver regarded as relevant.
3. Final iVEPs: acquired at the end of surgery with the patient still under anesthesia, before removing the surgical dressing.

Variations in the iVEP amplitude > 10% compared to baseline were considered significant. The changes in the iVEP amplitude occured during surgery and remained until the last acquisition made at the end of the procedure were defined as “stable.”

iVEP latency variations were also considered in the preliminary analysis. However, its changes were minimal, were not statistically significant and therefore were not further considered.

### Neuro-anesthetic protocol

The anesthetic technique used was TIVA (Total IntraVenous Anesthesia) with propofol and remifentanil both TCI (Target Controlled Infusion) [25, 29, 30]. Details are provided in Supplementary Methods. Bispectral index (BIS) was kept stable at values 45–50 throughout the VEP recording phase.

### Endoscopic endonasal approach (EEA)

Patients were operated on by a multidisciplinary team consisting of Otolaryngologist and Neurosurgeon with extensive experience in EEA. The technique used was the binostril transsphenoidal approach with posterior septostomy, as previously described [5, 12]. Details are provided in Supplementary Methods.

### Postoperative evaluation and follow-up

The first postoperative evaluation was performed 72 h after surgery: visual acuity and visual field testing were performed with the same modalities of baseline. The tests were then repeated at 3 months follow-up, establishing the final result of visual acuity and visual field for each patient, used for the statistical analysis.

Improved or worsened changes of at least 1/10 in visual acuity were considered significant. For visual field, variations of at least one campimetric quadrant were considered significant.

### Statistical analysis

We analyzed separately visual acuity, visual field, and iVEPs of each eye (*n* = 128). Continuous values were described using mean ± standard deviation (SD), categorical variables using absolute and relative frequencies. Correlation between continuous variables was performed by plotting regression lines and calculating the Spearman correlation coefficient. Comparison of continuous variables between groups was performed using the Mann–Whitney *U* test, and comparison of categorical variables was performed using the chi-square statistics adopting the Fisher exact test when appropriate. To assess the reliability of perioperative VEP variation in predicting visual change, we plotted ROC curves, calculated the area under the curve (AUC) and assessed the best cutoff. The latter was defined as the threshold value at which the Youden index (i.e., the difference between sensitivity and 1-specificity), has its maximum value. Analyses were performed using StatView ver 5.0 (SAS Institute, Cary, NC, USA) and MedCalc ver 20.015 (MedCalc Software Ltd, Osted, Belgium).

## Results

### Study population

**Fig. 2:**
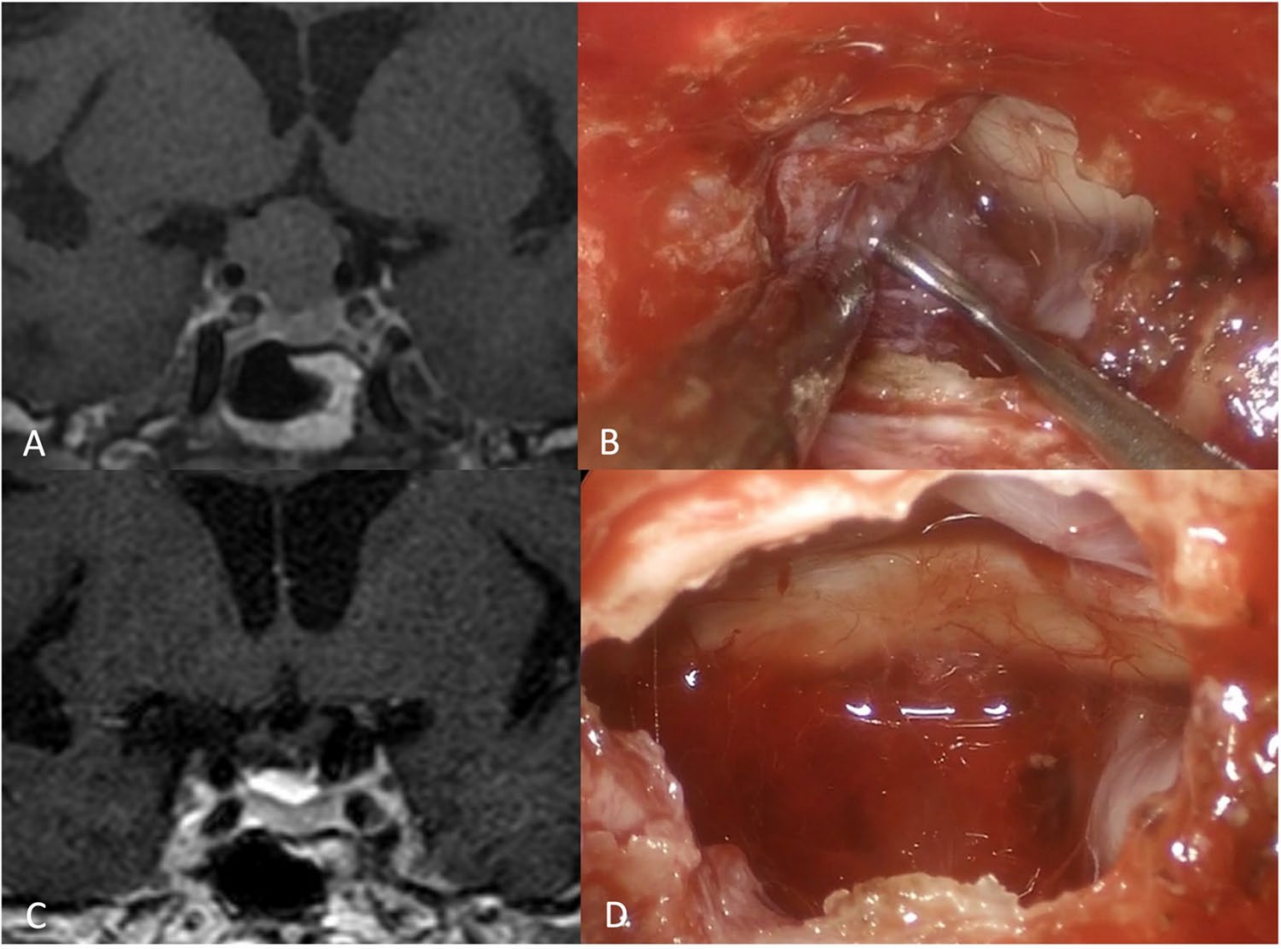
Diaphragm meningioma with optic chiasm compression and visual impairment (**A**, **C)**; surgical phases of tumor removal (**B**); and final optic chiasm decompression (**D**)

Sixty-four patients were included (Table 1). The mean age at diagnosis was 57.7 years (range 18–84); the male/female ratio was 31/33. The most frequent histological diagnoses were pituitary adenoma (29 cases, 45.3%), meningioma (12 cases, 18.7%), and craniopharyngioma (10 cases, 15.6%) (Fig. 2).

**Table 1:** Summary of patients’ characteristics

Patients (*n*)	64	
Age (median)	57.7	
Istology *n* (%)	Pituitary adenoma	29 (45.3%)
	Meningioma	12 (18.7%)
	Craniopharyngioma	10 (15.6%)
	Sellar/suprasellar cysts	5 (7.8%)
	Clival cordoma	3 (4.7%)
	Clival/sellar metastasis	2 (3.1%)
	Other	3 (4.7%)
Visual acuity impairment *n* (%)	55 (85.9%)	
Visual field impairment *n* (%)	49 (76.5%)

The preoperative visual function assessment revealed a reduction in vision in at least one of the two eyes in 55 patients (85.9%) and a reduction in the visual field in at least one of the two eyes in 49 patients (76.5%).

### Postoperative visual outcome

Out of 128 eyes of the study, visual acuity improved in 54 (42.2%), remained stable in 71 (55.5%) and worsened in 3 eyes (2.3%), with a maximum increase of 9/10 and a maximum decrease of 8/10.

Visual field improved in 65 eyes (53.3%) and remained stable in 57. In 6 eyes the visual field evaluation could not be carried out.

### Baseline iVEPs reliability: accordance to the preoperative vision

**Fig. 3:**
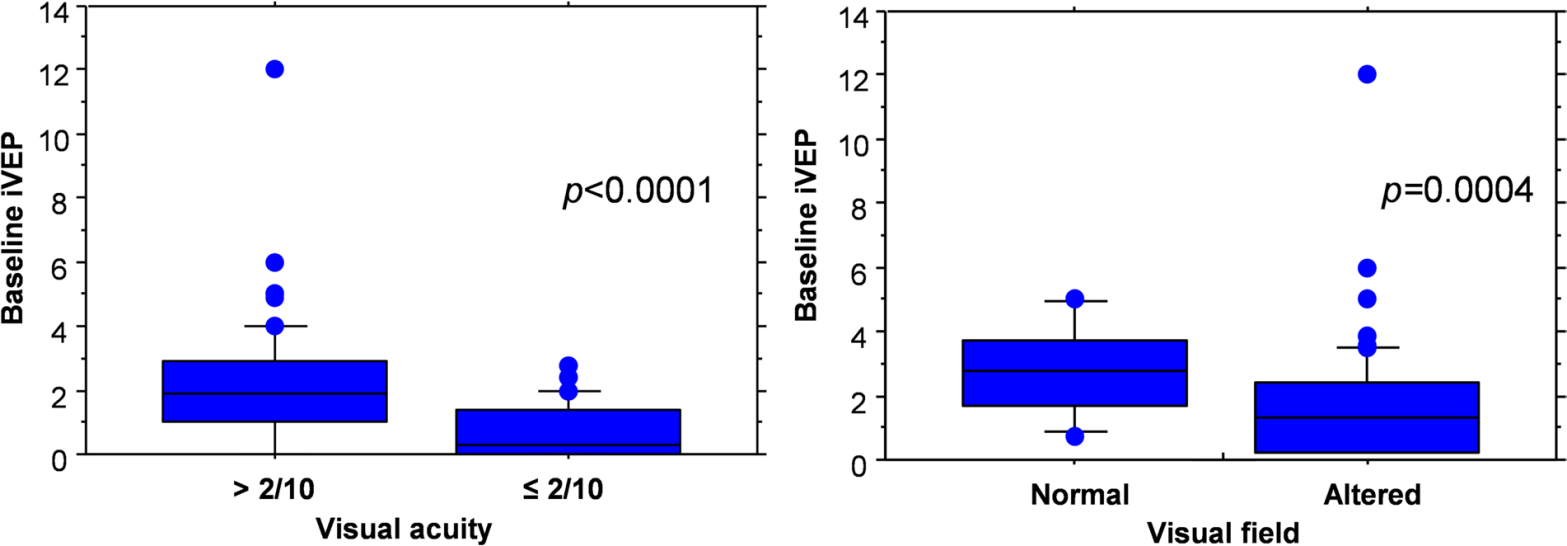
Baseline iVEPs and preoperative vision. Left. Box plot comparing baseline iVEPs in patients with preoperative visual acuity > 2/10 vs ≤ 2/10. Right. Box plot comparing baseline iVEPs in patients with any visual field impairment vs those with no impairment

Mean BIS value during the recording phase was 47.9 ± 3.2. The baseline iVEPs amplitude strictly correlated with the preoperative visual assessment. In detail, we found a significant correlation between preoperative visual acuity and iVEPs amplitude (*p* = 0.0010, Spearman correlation coefficient). Mean baseline iVEPs amplitude was significantly higher in patients with preoperative visual acuity > 2/10 than in patients with preoperative visual acuity ≤ 2/10 (2.1 ± 1.8 vs 0.7 ± 0.9, *p* < 0.0001, Mann–Whitney *U* test) (Fig. 3, left). Moreover, mean preoperative iVEPs amplitude was significantly higher in patients with normal preoperative visual field than in patient with altered preoperative visual field (2.6 ± 1.4 vs 1.6 ± 1.8, *p* = 0.0004, Mann–Whitney *U* test) (Fig. 3, right*)*.

### iVEPs and postoperative visual function

**Fig. 4:**
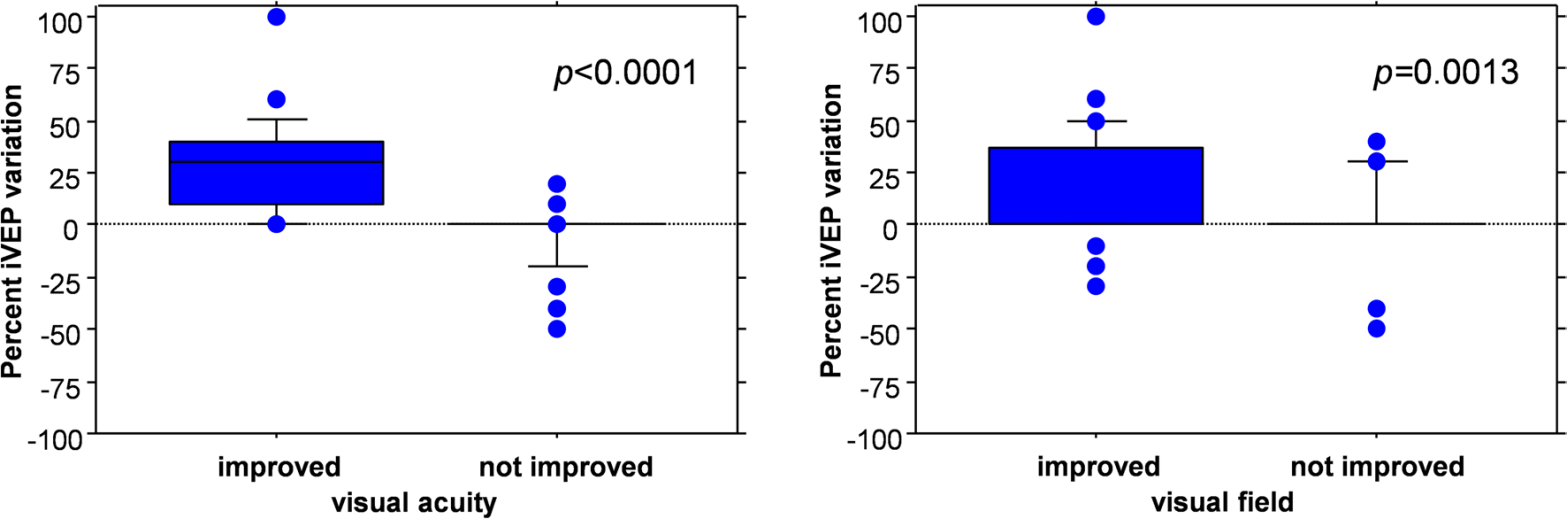
Postoperative iVEPs variation and postoperative vision. Left. Box plot comparing iVEPs variation in patients with improved vs not improved postoperative visual acuity. Postoperative iVEPs amplitude significantly increased in the former vs the latter patients (28.7% ± 22.9% vs − 4.9% ± 16.5%, *p* < 0.0001, Mann–Whitney *U* test). Right. Box plot comparing postoperative iVEPs variation in patients with improved vs not improved postoperative visual field. Postoperative iVEPs significantly increased in the former vs the latter patients (16.9% ± 26.6% vs 0.7% ± 21.8%, *p* = 0.0013, Mann–Whitney *U* test)

iVEPs variations were strongly correlated with the postoperative visual changes. In detail, we found a linear correlation between the changes of visual acuity and changes of VEP amplitude (*p* < 0.0001, Spearman correlation coefficient). Moreover, mean percent iVEPs variation was significantly higher in patients with improved visual acuity than in patients with stable or worsened visual acuity after surgery, and in patients with improved visual field than in those with stable or worsened visual field after surgery (*p* < 0.0001 and *p* = 0.0013, respectively; Mann–Whitney *U* test; Fig. 4).

### Sensitivity, specificity, and predictive value of iVEPs for visual function

**Fig. 5:**
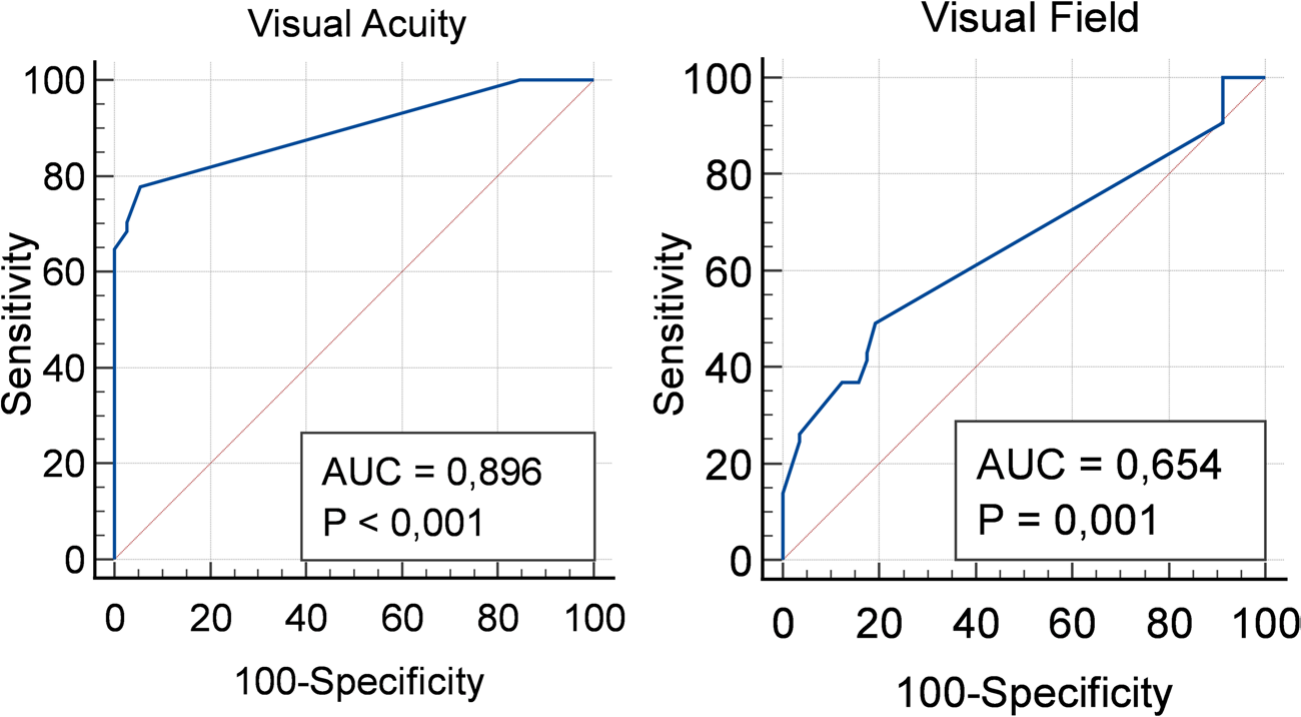
Left*.* ROC curve assessing the reliability of iVEPs variation in predicting visual acuity improvement. Right*.* ROC curve assessing the reliability of iVEPs variation in predicting visual field improvement

By ROC analysis, we found that the percent iVEPs variation was a good predictor of visual acuity improvement after surgery (AUC 0.896), with a best cutoff set at zero (Fig. 5, left). Conversely, ROC analysis showed that the percent iVEPs variation was a suboptimal predictor of visual field improvement after surgery (AUC 0.654): also, in the latter, the best cutoff was set at zero (Fig. 5, right).

We dichotomized the percent iVEPs variation using the identified best cutoff to establish the predictive positive and negative values of iVEPs variation for vision improvement after surgery. As concerns visual acuity improvement, predictive positive value was 91.3% and a predictive negative value was 85% (*p* < 0.0001, Fisher Exact test; Table 2). By limiting analysis to those patients with preoperative visual acuity < 10/10, we found a positive predictive value of VEP variation for visual improvement of 100%, whereas the negative predictive value dropped at 78.2% (*p* < 0.0001, Fisher exact test). In other words, an improvement of iVEPs predicts a visual acuity recovery in all cases.

**Table 2:** Positive and negative predictive value of iVEP for postoperative vision outcome

		Visual acuity		Visual field	
		Improved	Stable/worsened	Improved	Stable/worsened
iVEPs	Improved	42 (91.3%)	4 (8.7%)	32 (74.4%)	11 (25.6%)
	Stable/worsened	12 (15%)	68 (85%)	33 (41.8%)	46 (58.2%)

As expected, the positive and negative predictive values of iVEPs variation for visual field improvement were lower (74.4% and 58.2%, respectively, with *p* = 0.0006; Fisher exact test; Table 2). By limiting the analysis at those patients with preoperative reduced visual field, the positive predictive value raised to 86.5%, while the negative predictive value remained low (42.1%) (*p* = 0.0055, Fisher Exact Test). In other words, an iVEPs augmentation was associated to a postoperative visual field restitution, and patients with stable or reduce iVEPs could also have the chance of a postoperative visual field improvement.

## Discussion

### State of the art of techniques and reliability of intraoperative monitoring of VEPs

The VEPs, as widely described, allow an objective comprehensive evaluation of the function of the optic nerve, chiasm, and the whole visual pathway, without the needs of patient’s cooperation [10, 21, 31]. Furthermore, it has been proven that compression of visual pathway, as well as intrinsic lesions of the optic-chiasmatic apparatus, can influence VEPs, resulting in an increase in latency, as well as in a reduction of the amplitude of the wave potential [7, 26, 27, 31].

Nevertheless, conflicting data are reported on the intraoperative reliability of VEPs as predictors of visual outcome. Inconclusive results are probably affected by the inconsistency of anesthetic and monitoring techniques, particularly in older studies. The introduction of total intravenous anesthesia (TIVA) [27, 29, 30] and the simultaneous use of EEG monitoring during iVEPs acquisition [13] allowed more stable and reliable acquisitions during surgery. In Table 3, we resumed the findings of the most numerous clinical series: incompleteness of reported data and the consequent inconclusive results are evident [3, 4, 8, 13, 15, 17–19, 22, 23, 28].

**Table 3:** Report of sensitivity, specificity positive, and negative predictive values in studies on iVEPs in EEA

Author	Number of patients	Sensitivity	Specificity	Positive predictive value	Negative predictive value
Our results	64	77.8%	94.4%	91.3%	85%
Feng et al. (2019) [8]	42	25%	97%	50%	93%
Qiao et al. (2019) [22]	76	–	–	–	–
Toyama et al. (2018)	20	n/a	85%	n/a	100%
Nishimura et al. (2018) [19]	82	n/a	95%	n/a	100%
Kurozumi et al. (2017) [17]	19	–	–	–	–
Luo et al. (2015) [18]	46	n/a	96%	n/a	90%
Kamio et al.a (2014) [14]	33	100%	100%	100%	100%
Houlden et al.a (2014) [13]	10	–	–	–	–
Chung et al. (2012) [4]	53	–	–	–	–
Sasaki et al. (2010) [23]	28	88%	96%	64%	99%
Chacko et al. (1996) [3]	36	n/a	100%	n/a	100%

### Findings of the present study

#### Realization of a robust and reliable set-up for iVEP monitoring

We obtained a reliable and reproducible iVEP monitoring in all 64 included patients, regardless of clinical characteristics and type of pathology. Our clinical series is homogeneous in terms of neuro-anesthetic (all patients performed TIVA) and neurophysiological protocols (iVEPs, ERG, and EEG), approach (EEA) and surgical team (Neurosurgeon and ENT surgeon).

Notably, our study differs in the stimulation and recording techniques respect to the previous literature. In fact, the light power of the stimulus used in most of the published studies is 2000 lx with a stimulus duration of 20 ms and a stimulation frequency of 1 Hz, while in our study 3 white light LEDs were used with a power of 5500 lx each (total 16,500 lx for each eye) with a stimulus duration of 5 ms and a stimulation frequency of 1 Hz. In our experience, the high brightness and the shortness of the stimulus are important to obtain a recordable response from suffering optical pathways such as those examined; on the other hand, a stimulus of low and prolonged intensity is unlike to evoke recordable responses. Furthermore, most of the published series reported on the use of goggle glasses with red LEDs, which are less effective in retinal stimulation than the white light LEDs used in this study [3, 15, 17, 19, 23, 28]. Finally, in many papers simultaneous ERG monitoring was not carried out [4, 8, 13, 18, 22], which instead is an important parameter for verifying the effectiveness of the VEP visual stimulation performed [2, 9, 11, 15, 23, 24].

#### Baseline iVEPs values strongly correlate with preoperative visual function

Our results confirm that the basal iVEPs, and in particular the wave amplitude, significantly correlate with the preoperative visual status, both as regards visual acuity and visual field (Fig. 3). Such data confirm the reliability and robustness of the neuroanesthetic/neurophysiological protocols adopted.

#### Modifications of iVEPs strongly correlate to changes in visual function

Our results testify that stable variations of the iVEPs were strongly correlated to changes in postoperative vision, particularly concerning visual acuity (Fig. 4). Specificity and positive predictive value were remarkable high (94.4% and 91.3%, respectively) (Fig. 5 and Table 3).

An important point is to establish the ideal threshold to consider a stable modification of iVEP amplitude “significant.” In most studies, an iVEP with > 50% increase in amplitude from baseline is generally considered “improved” and an iVEP with > 50% decreased amplitude from baseline is worsened [4, 8, 17, 28]. This relatively high threshold of variation can be justified by the relative instability of the visual potentials or by the difficulty in interpreting more subtle modifications. Few studies used lower thresholds, with unclear results [22]. In the present study, we planned to consider significant any variation of VEP amplitude regardless of its extent (with a threshold set at 10% to account for background noise) but in relation to its stability over time. Our analysis demonstrates that an amplitude augmented of ≥ 10% was able to predict postoperative visual improvement with 100% reliability. On the other hand, we were not able to establish a statistical correlation between the worsening of iVEPs and visual worsening because of the small number of worsening observed: but what we can say is that a reduction of final iVEPs amplitude has been observed only in the 2 patients (3 eyes) that experienced a worsening of the vision post-surgery. One of the patients was harboring a giant and harsh relapsed nonfunctioning pituitary adenoma invading the posterior fossa: in this case removal was complete and worsening was probably due to injury of the vascular supply of the branches of the superior hypophyseal arteries supplying the chiasma; this patient accounted for 2 out of the 3 postoperative visual worsening in this series; the other patient had a stiff tuberculum sellae meningioma that was very strictly adherent to the left optic nerve; in this patient, because of iVEPs deterioration in the left eye during tumor removal, we decided to interrupt surgery since a satisfied decompression had been already obtained.

### Strengths and limitations of the present study

We homogeneously and prospectively collected and analyzed data from a single tertiary skull base reference center; moreover, we obtained statistically robust evidence on the positive predictive role of iVEP in predicting patients’ visual status. These considerations build up the strong points of the study.

As limitations, the neurophysiologic and anesthesiologist setting can be somewhat demanding and needs to be validated in multicenter studies to assess generalizability. Moreover, the predictive value of transient iVEPs worsening could not be rigorously assessed (explaining the absence of correlation between mean percent iVEPs variation and visual function in patients with worsened visual acuity after surgery), nor was a correlation between a precise surgical step (dural opening, tumor debulking, and so on) and corresponding iVEPs variation.

It is worth to note that our results were obtained in a particular setting with an EEA and cannot be translated in other surgical approaches. It is in fact our experience that by using the same intraoperative protocol during intervention performed with a transcranial approach the methods can be less reliable due to displacement of stimulating ipsilateral goggle induced by the surgical maneuvers as frontal skin detachment necessary for the flap and craniotomy.

## Conclusion

The present study demonstrates that iVEPs can be reliably recorded during EEA. We set up a reproducible method and identified the cutoff values of significance for the variations in amplitude that occur during surgery. Further studies are necessary to demonstrate and confirm the sensitivity of this neurophysiological tool in guiding live surgery.

### Supplementary Information

Below is the link to the electronic supplementary material.

Supplementary file1 (DOCX 19 KB)

## Abbreviations

iVEPs: Intraoperative visual evoked potentials
EEA: Endonasal endoscopic approach
SSEP: Somatosensory evoked potentials
MEP: Motor evoked potentials
VEPs: Visual evoked potentials
EEG: Electroencephalogram
ENT: Ear, nose, and throat
ERG: Electroretinogram
TIVA: Total IntraVenous Anesthesia
TCI: Target Controlled Infusion
BIS: Bispectral index
ABB: Acid base balance
CSF: Cerebro-spinal fluid
SD: Standard deviation
AUC: Area under the curve

## References

[1] CBTRUS 2002 statistical report: primary brain tumors in the United States, 1995–1999. Central Brain Tumor Registry of the United States. Available from: http://www.cbtrus.org

[2] Cedzich C, Schramm J, Fahlbusch R (1987). Are flash-evoked visual potentials useful for intraoperative monitoring of visual pathway function?. Neurosurgery.

[3] Chacko AG, Babu KS, Chandy MJ (1996). Value of visual evoked potential monitoring during trans-sphenoidal pituitary surgery. Br J Neurosurg.

[4] Chung SB (2012). Intraoperative visual evoked potential has no association with postoperative visual outcomes in transsphenoidal surgery. Acta Neurochir (Wien).

[5] D'alessandris QG, Rigante M, Mattogno PP, La Rocca G, Romanello M, Auricchio AM, Bevacqua G, Fraschetti F, Giordano M, Di Bonaventura R, Pallini R, Anile C, Olivi A, Lauretti L (2020). Impact of 4K ultra-high definition endoscope in pituitary surgery: analysis of a comparative institutional case series. J Neurosurg Sci.

[6] Doglietto F, Prevedello DM, Jane JA, Han J, Laws ER (2005). Brief history of endoscopic transphenoidal surgery–from Philipp Bozzini to the first world congress of endoscopic skull base surgery. Neurosurg Focus..

[7] Feinsod M, Selhorst JB, Hoyt WF, Wilson CB (1976). Monitoring optic nerve function during craniotomy. J Neurosurg.

[8] Feng R, Schwartz J, Loewenstern J, Kohli K, Lenina S, Ultakan S, Iloreta AM, Govindaraj S, Bederson J, Banik R, Shrivastava R (2019). The predictive role of intraoperative visual evoked potentials in visual improvement after endoscopic pituitary tumor resection in large and complex tumors: description and validation of a method. World Neurosurg..

[9] Flanagan J, Harding G (1988). Multi-channel visual evoked potentials in early compressive lesions of the chiasm. Doc Ophthalmol.

[10] Gott PS, Weiss MH, Apuzzo M, Van Der Meulen JP (1979). Checkerboard visual evoked response in evaluation and management of pituitary tumors. Neurosurgery.

[11] Gutzwiller EM (2018). Intraoperative monitoring with visual evoked potentials for brain surgeries. J Neurosurg.

[12] Hadad G, Bassagasteguy L, Carrau RL (2006). A novel reconstructive technique after endoscopic expanded endonasal approaches: vascular pedicle nasoseptal flap. Laryngoscope.

[13] Houlden DA (2014). Intraoperative flash VEPs are reproducible in the presence of low amplitude EEG. J Clin Monit Comput.

[14] Jashek-Ahmed F, Cabrilo I, Bal J, Sanders B, Grieve J, Dorward NL, Marcus HJ (2021). Intraoperative monitoring of visual evoked potentials in patients undergoing transsphenoidal surgery for pituitary adenoma: a systematic review. BMC Neurol.

[15] Kamio Y, Sakai N, Sameshima T (2014). Usefulness of intraoperative monitoring of visual evoked potentials in transsphenoidal surgery. Neurol Med-Chir.

[16] Kodama K, Goto T, Sato A, Sakai K, Tanaka Y, Hongo K (2010). Standard and limitation of intraoperative monitoring of the visual evoked potential. Acta Neurochir (Wien).

[17] Kurozumi K (2017). Simultaneous combination of electromagnetic navigation with visual evoked potential in endoscopic transsphenoidal surgery: clinical experience and technical considerations. Acta Neurochir (Wien).

[18] Luo Y (2015). Clinical utility and limitations of intraoperative monitoring of visual evoked potentials. PLoS One.

[19] Nishimura F (2018). Efficacy of the visual evoked potential monitoring in endoscopic transnasal transsphenoidal surgery as a real-time visual function. Neurol India.

[20] Odom JV, Bach M, Brigell M, Holder GE, McCulloch DL, Mizota A (2016). ISCEV standard for clinical visual evoked potentials: (2016 update). Doc Ophthalmol.

[21] Petersen J (1984). Objective determination of visual acuity by visual evoked potentials. Spec Tests Vis Funct.

[22] Qiao N, Song M, Ye Z, He W, Ma Z, Wang Y, Zhang Y, Shou X (2019). Deep learning for automatically visual evoked potential classification during surgical decompression of sellar region tumors. Transl Vis Sci Technol.

[23] Sasaki T (2010). Intraoperative monitoring of visual evoked potential: introduction of a clinically useful method. J Neurosurg.

[24] Sato A (2016). Interpretation of the causes of instability of flash visual evoked potentials in intraoperative monitoring and proposal of a recording method for reliable functional monitoring of visual evoked potentials using a light-emitting device. J Neurosurg.

[25] Schnider TW, Minto CF, Gambus PL, Andresen C, Goodale DB, Shafer SL, Youngs EJ (1998). The influence of method of administration and covariates on the pharmacokinetics of propofol in adult volunteers. Anesthesiology.

[26] Semela L, Hedges TR, Vuong L (2007). Serial multifocal visual evoked potential recordings in compressive optic neuropathy. Ophthalmic Sur Lasers Imaging Retina.

[27] Tanaka R, Tanaka S, Ichino T, Ishida T, Fuseya S, Kawamata M (2020). Differential effects of sevoflurane and propofol on an electroretinogram and visual evoked potentials. J Anesth.

[28] Toyama K, Wanibuchi M, Honma T, Komatsu K, Akiyama Y, Mikami T, Mikuni N (2020). Effectiveness of intraoperative visual evoked potential in avoiding visual deterioration during endonasal transsphenoidal surgery for pituitary tumors. Neurosurg Rev.

[29] Uribe AA (2017). Comparison of visual evoked potential monitoring during spine surgeries under total intravenous anesthesia versus balanced general anesthesia. Clin Neurophysiol.

[30] Wiedemayer H (2003). Visual evoked potentials for intraoperative neurophysiologic monitoring using total intravenous anesthesia. J Neurosurg Anesthesiol.

[31] Wilson W, Kirsch W, Neville H, Stears J, Feinsod M, Lehman R (1976). Monitoring of visual function during parasellar surgery. Surg Neurol.

[32] Zada G, Du R, Laws ER (2011). Defining the “edge of the envelope”: patient selection in treating complex sellar-based neoplasms via transsphenoidal versus open craniotomy. J Neurosurg.

