# Supplementary material for: Reliability of intraoperative visual evoked potentials (iVEPs) in monitoring visual function during endoscopic transsphenoidal surgery
Source: Acta Neurochir (Wien). 2023 Sep 21;165(11):3421–9. doi: 10.1007/s00701-023-05778-1 (PMC10624729; doi:10.1007/s00701-023-05778-1)
Supplement: Supplementary file 1 — Supplementary file1 (DOCX 19 KB) [file 701_2023_5778_MOESM1_ESM.docx]

**Reliability of intraoperative visual evoked potentials (iVEPs) in monitoring visual function during endoscopic transsphenoidal surgery.**

**Supplementary Methods**

*Neuro-anesthetic Protocol*

The anesthetic technique used is based on TIVA (Total IntraVenous Anesthesia) with propofol and remifentanil both TCI (Target Controlled Infusion)[16,20]. TCI modality is well suited to neurosurgical procedures, allowing for more precise control over the effects of anesthetic drugs on neurophysiological parameters. All patients received an oral 0.2 mg/kg midazolam dose as premedication approximately 1 hour before surgery. No other sedative or centrally acting agent was administered prior to the induction of anesthesia. Anesthesia was induced with a loading dose of remifentanil 2-3 ng/ml in continuous infusion (Alaris PK) based on the pharmacokinetic model of Minto, followed 5 minutes with 3- 3.5 µg /ml propofol as dose of induction. The TCI system used for target-controlled delivery of propofol was set up on the Alaris PK pump and was based on Schnider's pharmacokinetic model [29]. Endotracheal intubation was facilitated by 0.08 mg/kg vecuronium bromide; no further doses of muscle relaxants were administered during surgery. The lungs were mechanically ventilated with a 45% O2 mixture in air, to maintain end-tidal CO2 (ETCO2) concentrations at 30-35 mmHg during surgery. Anesthesia was maintained with remifentanil 4-5 ng/ml and propofol in a range of 2.5 to 3.0 μg/ml according to the physiological parameters of the patients and BIS (Bispectral Index) monitoring, to obtain a constant level of anesthesia. In the event of signs of light anesthesia (increased heart rate or MAP [mean arterial pressure]> 15% from baseline, BIS values over 50-60), the infusion rate of remifentanil was increased to 5-6ng/mL and propofol up to 4 µg/ml. During the recording of evoked potentials, a stable anesthetic status was maintained without further administration of bolus drugs; BIS values were kept stable at 45-50 throughout the VEP recording phase for all included patients. Routine anesthetic monitoring included ECG, heart ratio, SpO2, invasive blood pressure, ETCO2, pharyngeal (core) and skin temperature, BIS. Arterial blood samples were analyzed periodically to keep all ABB (Acid Base Balance) parameters within the normal range. At the end of the surgical procedure, all patients awakened within 15-30 minutes of stopping the TIVA.

*Surgical technique*

Patients were operated on by a multidisciplinary team consisting of Otolaryngologist and Neurosurgeon with extensive experience in EEA. The technique used was the binostril trans-sphenoidal approach with posterior septostomy and harvesting of a modified "rescue flap" and / or a pedunculated nasoseptal flap according to Hadad-Bassagasteguy, carefully preserving the septal branch of the sphenopalatine artery which supplies the flap [12]. In cases of suprasellar extension of the lesion, removal of the tuberculum sellae or part of the spheno-ethmoidal planum was carried out in order to have the widest possible view of the suprasellar region and of the optic-chiasmatic complex, to perform the most effective and safe possible decompression. After removal of the lesion, sellar floor reconstruction was carried out with Gelfoam, fibrin glue and autologous septal bone/cartilage; alternatively, in cases of evidence of intraoperative cerebro-spinal fluid (CSF) leakage, autologous adipose tissue harvested from the abdominal subcutaneous was positioned, covered by the previously prepared nasoseptal pedunculated flap [12].

The endoscopic instruments used were the UHD System (Visera 4K UHD, Olympus, Tokyo, Japan) consisting of a camera (CH-S400, Olympus) equipped with a 300 W Xenon light source (CLV-S400, Olympus), a control (OTV-S400, Olympus) and a dedicated ultra-HD 55 '' main screen (LMD-X550S, Olympus).

0°, 30° and 45° optics, diameter 4 mm, length 18 cm (Olympus, Tokyo, Japan) were used for all the procedures performed.
